# Supplementary material for: Point-of-care creatinine-based eGFR (StatSensor) in detecting kidney dysfunction (KD) among people living with HIV in Tanzania
Source: PLoS One. 2025 Sep 11;20(9):e0331969. doi: 10.1371/journal.pone.0331969 (PMC12425315; doi:10.1371/journal.pone.0331969)
Supplement: Attachment — Submitted filename: Reviewers comments.docx [file pone.0331969.s005.docx]

| **Response to reviewers comments** | | |
| --- | --- | --- |
| **Comment** | | **Response** |
| **Reviewer 2** | |  |
| 1 | The bland Altman plot clearly when truncated shows a positive bias for eGFR at lower ends up to 60 ml/min. This explains the 4 normal mis-classification, however at higher eGFR there is a negative bias explaining the 16 cases of miss classification. This explanation must be highlighted in the results discussion. | Thank you for the insightful comment. We have now clarified the interpretation of the Bland–Altman plot in the **Discussion** sections and added the following details. **Interestingly, the bias appeared to vary across the eGFR range. At lower eGFR values (<60 mL/min/1.73 m²), there was a mild positive bias, contributing to the misclassification of a few normal cases as abnormal. Conversely, at higher eGFR values, the StatSensor tended to underestimate eGFR, consistent with the overall negative bias, leading to some misclassification of normal as reduced kidney function. (PAGE7)** |
| **Reviewer 1** | |  |
| 1 | Including the creatinine data would add value to this publication (at least the central data for each method) | We have added the creatinine data in table 1 |
| 2 | **A.** Legend for Table 3 (line 183/184) still refers to "laboratory enzymatic eGFR". Should be "Jaffe" | Thank you for pointing this out. The term has been corrected to “Jaffe” in the legend of Table 3 (Page 6). |
|  | **B.** Line 154, please add the median (IQR) data for the point of care eGFR results either in Table 1 or in the paragraph describing Table 2 data. | We have now included the median (IQR) for the StatSensor eGFR values in the Results section and added it to Table 1 (Page 5). |
|  | **C.** eGFR graphs: * Bland-Altman graph: The TAE error limits have been plotted incorrectly. The TAE is a percentage, whereas the results are plotted in ml/min/1.73m2. See attached example. * If possible, please add the TAE limits to the ordinary linear regression graph as well. See attached example. | We appreciate this clarification. The Bland–Altman plot has been revised to reflect the Total Allowable Error (TAE) in percentage format as recommended. We also added TAE limits to the regression graph (Figures 2 and 3 revised). |
|  | **D.** Under the RESULTS and DISCUSSION sections: * Line 174 – 176: The linear regression relationship demonstration should be described as the equation (y=0.903x+3.82). In the next line, it is incorrectly stated that a positive bias of 4.36 ml/min/1.73m2 is present – it appears to be a negative bias (which correlates with the linear regression equation).  The rest of the discussion and conclusions should be adjusted around this small negative bias and over-calling of decreased kidney function. | We have revised the relevant lines in the **Results** and **Discussion** sections to correctly describe the direction of bias. Specifically, the bias is now clearly described as **negative,** consistent with the regression equation. The discussion has been adjusted to reflect the implications of a negative bias on clinical decision-making and misclassification.  PAGE 7 |
|  | **E.** Consider changing the title to state "Point-of-care creatinine based eGFR (StatSensor) in detecting kidney dysfunction (KD) among people living with HIV in Tanzania" | We have revised the title to; “Point-of-care creatinine-based eGFR (StatSensor) in detecting kidney dysfunction among people living with HIV in Tanzania.” |
|  | **F.**  Adjust abstract according to above changes. In the Results section: An example would be: The StatSensor eGFR correlated with Roche Jaffe method-derived eGFR (y=0.903x+3.82; r2=0.813), with a mean negative bias of 4.36 ml/min/1.73m2. | The **Abstract** has been revised to include the regression equation (y = 0.903x + 3.82; R² = 0.813) and mention the negative bias (-4.36 ml/min/1.73 m²), consistent with the main text **(Page 2).** |
